# Effect of a new irrigant solution containing glycolic acid on smear layer removal and chemical/mechanical properties of dentin

**DOI:** 10.1038/s41598-020-64450-1

**Published:** 2020-04-30

**Authors:** Débora Pereira Diniz Correia Barcellos, Ana Paula Farina, Ramiro Barcellos, Matheus Albino Souza, Márcia Borba, Ana Karina Bedran-Russo, Yuri Dal Bello, Cristina De Mattos Pimenta Vidal, Doglas Cecchin

**Affiliations:** 1 Department of Restorative Dentistry, College of Dentistry, University of Passo Fundo, Passo Fundo, RS Brazil; 2 Department of Restorative Dentistry, College of Dentistry, University of Illinois at Chicago, Chicago, IL USA; 3 Department of Operative Dentistry, College of Dentistry, University of Iowa, Iowa City, IA USA

**Keywords:** Dental materials, Dental treatments, Endodontics

## Abstract

The objective of this study was to evaluate the effects of glycolic acid (GA) (with pH 1.2 and 5) and ethylenediaminetetraacetic acid (EDTA) on the chemical and mechanical properties of dentin to investigate the potential use of GA as final irrigant in the root canal therapy. Specifically, changes in microhardness, smear layer removal, erosion, mineral content distribution, apatite/collagen ratio and flexural strength of mineralized dentin treated with GA were assessed. Saline solution was used as a negative control. Knoop microhardness (KHN) was measured on the root canal lumen of root segments. Dentin beams were used for 3-point flexural strength (σ) test. Scanning electron microscopy (SEM) images of root sections were obtained for evaluation of smear layer removal and dentin erosion on root segments and energy dispersive X-ray spectroscopy (EDS) was used for mineral content distribution. The apatite/collagen ratio (A/C) in dentin powder were examined by Fourier transform infrared (FTIR) spectroscopy. KHN, σ and A/C results were statistically analyzed with ANOVA and Tukey tests (α = 0.05). Smear layer and dentin erosion scores were analyzed with Kruskal-Wallis and Dunn tests (α = 0.05). Root dentin treated with EDTA and GA presented similar KHN regardless of the pH (p > 0.05). However, KHN was significantly reduced in EDTA and GA groups when compared to control group (p<0.001). GA showed the same ability to remove the smear layer and to cause dentin erosion as EDTA. EDS results showed that the GA and EDTA solutions did not alter the dentin mineral content distribution. The apatite/collagen ratio reduced with all irrigant solution and was the lowest with GA pH 5 (p<0.001), while σ was not significantly affected by the experimental solutions (p = 0.559). It can be concluded that GA has similar ability to remove the smear layer than EDTA. GA does not affect negatively the chemical/mechanical properties and it does not increase dentin erosion. The use of GA with low pH seems to promote less change in collagen/apatite ratio, but further studies are needed to establish an ideal clinical protocol. Therefore, this study supports the potential use of GA as an alternative final irrigation solution for root canal preparation.

## Introduction

The main purpose of the root canal preparation is cleaning and disinfection by using endodontic files and root canal irrigants^1^. However, this step results in the deposition of a smear layer on the root canal walls. The presence of the smear layer keeps bacteria and their byproducts in the root dentin, hinders the penetration of intracanal disinfectants and cements into the dentinal tubules, and decreases the sealing capacity of root canal sealers^2^. Therefore, smear layer removal during chemical and mechanical preparation of root canals has been proposed by the use of final irrigants. Different final irrigant solutions such as citric acid, MTAD (mixture of Doxycycline, citric acid and a detergent), etidronate and ethylenediaminetetraacetic acid (EDTA) are used for removal of the smear layer, being the EDTA still the most widely well-known in endodontics^3^.

However, an effective final irrigant solution must act only on superficial dentin removing the smear layer without causing damage to the internal portion of the root dentin. Although EDTA has good capacity of smear layer removal^3^, it has unfavorable features such as denaturation of collagen fibrils^4^ and peritubular and intertubular dentin erosion when used for more than three minutes^3^. These mineral changes in root canal dentin could have an effect on the adhesive properties of the dentin surface and reduce root canal sealing^5^. Furthermore, extrusion of EDTA solution beyond the apical foramen should be avoided because of its cytotoxicity^6^. Another important factor is that EDTA is produced on an industrial scale from ethylenediamine, formaldehyde, and sodium cyanide leading to the formation of contaminants that are considered some of the important pollutants dispensed in water^7^. Thus, EDTA plays a part in aquatic toxicity and may cause chronic effects, including the disequilibrium of body calcium in other organisms^8^. The investigation of alternative final irrigants that are biocompatible, effective in removing the smear layer without causing damage to the root dentin structure and properties is needed to ensure success of the root canal therapy.

The glycolic acid (GA) is an alpha hydroxy acid (AHA) extracted from sugar cane and other sweet vegetables. It is uncolored, odorless, has only two carbons in its molecular structure, and can be easily dissolved in water. GA is commonly used in dermatology for applications that range from skin moisturizing to deep chemical peeling, a common esthetic procedure. As the smallest AHA, GA has great penetration potential and its absorption on skin and mineral surfaces is faster than other AHA^9^. GA is found in concentrations of 2 to 10% in dermocosmetics and used in higher concentrations for chemical peeling (from 20 to 70%)^10^. In dentistry, recent studies showed GA to be suitable for enamel and dentin etching in restorative procedures and as efficient as EDTA in removing smear layer from root canals walls^11,12^. In addition, cytotoxic results indicated that GA has less toxic effects on fibroblasts when compared to EDTA^13^. Furthermore, GA showed pH stability for 90 days when stored at 4, 25 and 37 °C, which may facilitate its clinical use in endodontics, and did not promote alterations in dentin flexural strength^14^. Due to its positive characteristics, and considering the need for biologically compatible endodontic materials and substances, GA may be a suitable agent to remove the smear layer from the root canal walls with minimal negative biological effects.

Previously published studies from our research group suggest the use of GA as a final irrigant in the root canal therapy, since it demonstrated ability to remove the smear layer without negatively affecting the dentin properties when used at low pH (around 2)^13,14^. However, its application in endodontics relies on the establishment of a clinical protocol that details the concentration, pH, and irrigation time. The objective of the present *in vitro* study was to evaluate the effects of irrigation with 17% GA used with different pH (1.2 or 5.0) compared to 17% EDTA (neutral pH) on their capacity of smear layer removal from the root canals, changes in erosion, microhardness, mineral content distribution, apatite/collagen ratio and flexural strength of mineralized dentin. The tested null hypotheses were: (1) the 17% EDTA and 17% GA have similar capacity to remove the smear layer in all root canal thirds, regardless of the pH values of GA (1.2 or 5.0); and (2) there is no significant change on chemical and mechanical properties of root dentin when using 17% EDTA or 17% GA, regardless of the pH values.

## Results

**Figure 1:**
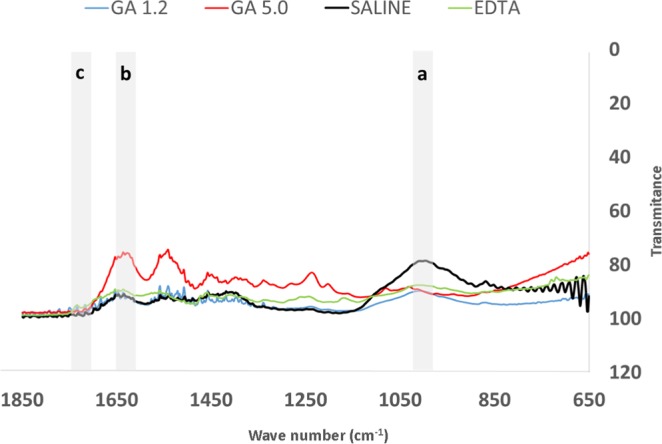
FTIR spectra of dentin powder after irrigation with the experimental solutions for 1 min. GA 1.2: glycolic acid at pH 1.2. GA 5.0: glycolic acid at pH 5.0. Saline: saline solution used in the control group. EDTA: ethylenediaminetetraacetic acid. Phosphate (**a**), amide I (**b**), and (**c**) peaks were used to obtain the apatite/collagen ratio.

**Figure 2:**
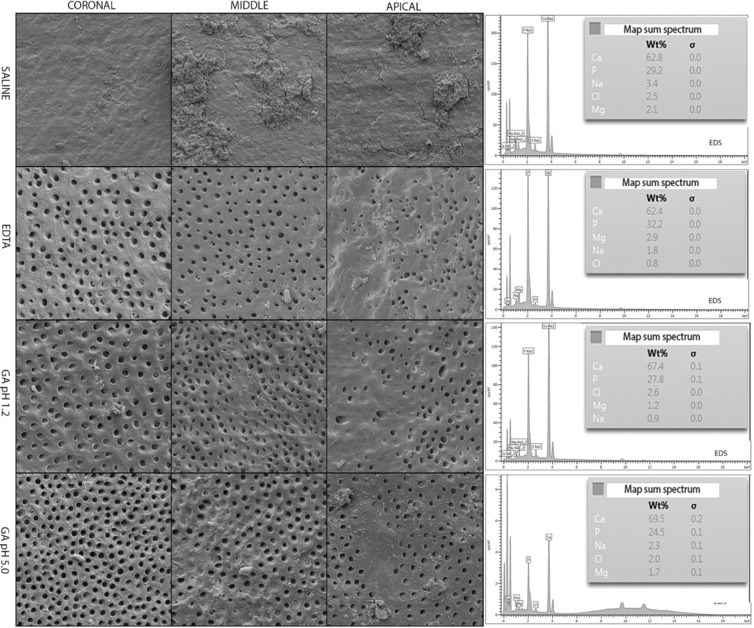
Representative scanning electron micrographs of all groups and root thirds and EDS results with the respective atomic ratios (at. %) of calcium (Ca), phosphorus (P), sodium (Na), chlorine (Cl), and magnesium (Mg).

Tables 1, 2 and 3 and Figs. 1 and 2 presented as follows depict the results from the systematic evaluation of effects of GA and EDTA on dentin chemical and mechanical properties and ability to remove the smear layer.

**Table 1:** Microhardness, Apatite/Collagen Ratios and Flexural Strength Values.

Groups	KHN	Flexural strength (MPa)	Apatite/collagen ratio
Saline (negative control)	40.3 ± 3.7^a^	3.4 ± 0.8^a^	2.7 ± 0.1^a^
2.5% NaOCl + 17% EDTA	21.2 ± 4.5^b^	4.1 ± 1.2^a^	0.9 ± 0.0^b^
2.5% NaOCl + 17% GA pH 1.2	24.0 ± 2.5^b^	3.7 ± 1.6^a^	1.0 ± 0.1^b^
2.5% NaOCl + 17% GA pH 5	25.1 ± 3.7^b^	4.1 ± 1.5^a^	0.3 ± 0.0^c^

NaOCl: sodium hypochlorite. EDTA: ethylenediaminetetraacetic acid. GA: glycolic acid.

Means followed by different letters in the same column present statistically significant difference (p < 0.001).

**Table 2:** Median (Md) and first (Q_1_) and third (Q_3_) quartiles of the scores of smear layer removal for the experimental groups.

EXPERIMENTAL GROUP	CORONAL			MIDDLE			APICAL			p value
	Md	Q_1_	Q_3_	Md	Q_1_	Q_3_	Md	Q_1_	Q_3_	
Saline	5.0 ^a A^	5.0	5.0	5.0 ^a A^	5.0	5.0	5.0 ^a A^	5.0	5.0	1.000
2.5% NaOCl + 17% EDTA	2.0 ^b B^	2.0	2.5	2.0 ^b AB^	2.0	2.5	3.0 ^b A^	2.0	3.0	0.007
2.5% NaOCl + 17% GA pH 1.2	1.5 ^b B^	1.0	2.0	2.0 ^b AB^	1.5	3.0	3.0 ^b A^	2.0	3.0	<0.001
2.5% NaOCl + 17% GA pH 5.0	2.0 ^b B^	2.0	2.0	2.0 ^b AB^	2.0	3.0	3.0 ^b A^	2.0	3.0	0.009
P value	<0.001	<0.001	<0.001							

NaOCl: sodium hypochlorite. EDTA: ethylenediaminetetraacetic acid. GA: glycolic acid.

*Medians followed by different lowercase letters in the same column are statistically different (p < 0.001). *Medians followed by different uppercase letters in the same row are statistically different (p < 0.05).

**Table 3:** Median (Md) and first (Q_1_) and third (Q_3_) quartiles of the erosion scores of the experimental groups.

EXPERIMENTAL GROUP	CORONAL			MIDDLE			APICAL			p value
	Md	Q_1_	Q_3_	Md	Q_1_	Q_3_	Md	Q_1_	Q_3_	
Saline	0.0 ^b A^	0.0	0.0	0.0 ^b A^	0.0	0.0	0.0 ^b A^	0.0	0.0	1.000
2.5% NaOCl + 17% EDTA	1.0 ^a A^	0.5	1.0	1.0 ^a A^	1.0	1.0	0.5 ^a A^	0.0	1.0	0.133
2.5% NaOCl + 17% GA pH 1.2	1.0 ^a A^	1.0	1.0	1.0 ^a AB^	0.0	1.0	0.5 ^a B^	0.0	1.0	0.006
2.5% NaOCl + 17% GA pH 5.0	1.0 ^a A^	1.0	1.0	1.0 ^a AB^	0.0	1.0	0.0 ^ab B^	0.0	1.0	0.010
P value	<0.001	<0.001	0.002							

NaOCl: sodium hypochlorite. EDTA: ethylenediaminetetraacetic acid. GA: glycolic acid.

*Medians followed by different lowercase letters in the same column are statistically different (p < 0.001). *Medians followed by different uppercase letters in the same row are statistically different (p < 0.05).

### Dentin microhardness, flexural strength and apatite/collagen ratio

The mean and standard deviation of the KHN values, flexural strength and apatite/collagen ratio of the experimental groups are presented in Table 1. There was a significant difference in KHN between the experimental groups (p < 0.001). Dentin irrigation with GA or EDTA resulted in statistically significant lower KHN than the control group (irrigated with saline solution). There was no statistically significant difference in KHN among EDTA and GA groups. Regarding flexural strength, there was no statistically significant difference among all groups (p = 0.559).

Figure 1 shows the FTIR spectra for the control, EDTA and GA groups used to obtain the apatite/collagen ratio. The apatite/collagen proportion significantly decreased after the irrigation with all experimental solutions, as the results obtained were statistically lower than the control group (no irrigation) (p<0.001). The lowest values were obtained for GA at pH 5.0 while EDTA and GA at pH 1.2 demonstrated statistically similar results (Table 1).

### Smear-layer removal, dentin erosion and mineral content distribution

Table 2 presents the smear layer scores of each root third (coronal, middle and apical) for each irrigant solution and saline (control). There was statistical significant difference in the scores between the coronal and apical thirds for EDTA, GA pH 1.2 and GA pH 5, with a higher score for the apical third. All irrigant solutions were able to remove partially the smear layer, with no statistical significant difference between EDTA and GA at different pH values for any of the root thirds evaluated.

The erosion scores for the experimental groups are summarized in Table 3. The saline solution could not remove the smear layer, and the dentin tubules were obliterated; hence, it was not viable to observe whether there was dentin erosion in this group. The erosion scores between the solutions were compared at each root third separately. EDTA and GA solutions at both pH values showed similar erosion scores, regardless of the root third evaluated. Additionally, when the different thirds were compared within the same irrigant solution, there was statistical significant difference between the coronal and apical thirds (p < 0.001).

Figure 2 demonstrates the representative SEM images of root canal walls and the atomic ratio (at. %) of Ca, P, Na, Cl, and Mg after the use of final irrigation solutions along the various thirds of the root canal walls. The SEM images show that in the coronal and middle thirds, the dentinal tubules were wide open on specimens treated with all the three experimental solutions. In the apical third, it is possible to observe a smaller number of open dentinal tubules. Samples treated with saline solution (control) presented heavy smear layer deposition in all the thirds of the root canal. According to the results of the EDS analysis, no major changes were observed in the chemical composition of dentin when comparing the different irrigant solutions.

## Discussion

In order to evaluate whether the irrigation with GA at acidic or neutral pH is effective in removing the smear layer and its potential effects on dentin surface integrity and chemical/mechanical properties when compared to EDTA, the pH values of 1.2 and 5.0 for GA were tested in the present study. The ability to remove the smear layer of EDTA was similar to GA at both pH values in each root third evaluated, so the first null hypothesis was accepted. Considering the chemical and mechanical properties evaluated in the present study, EDTA and GA irrigants caused significant reduction on the dentin microhardness, showed similar potential to promote erosion of root dentin walls, and no clear differences were noted in mineral composition on dentin surface. However, when comparing the collagen/apatite ratio, the most significant change was observed for GA at pH 5. Therefore, the second null hypothesis was rejected.

The decrease of dentin microhardness values promoted by EDTA compared to control (saline) is in agreement with other studies^13,15^. EDTA has the ability to combine with calcified components of dentin through a chelating mechanism resulting in demineralization and softening of tissue^3,15^. GA also showed significant reduction in microhardness than the control group, with similar changes regardless of the solution pH. GA has shown potential to demineralize dentin and remove smear layer in previous studies. When used as an etchant for smear layer removal prior to application of etch-and-rinse adhesive system, GA showed decrease of dentin microhardness comparable to phosphoric acid and ability to promote adequate bond strength^11^. In the same previously published study, GA was less aggressive in promoting enamel demineralization than PA^11^. Overall, irrigant solutions that present ability to remove the smear layer facilitate the access and action of endodontic instruments until the apical foramen, which is critical in narrow and calcified root canals^15^, but may cause reduction of dentin microhardness. Nevertheless, this reduction caused by the final irrigants does not seem to be detrimental to the fracture resistance of endodontically treated teeth^16^ when these solutions are used for a short period inside the root canal. For example, the resistance to fracture of endodontically treated roots significantly decreased when 17% EDTA persisted for 10 minutes or more inside the root canal^17^. Different irrigation time with GA should be explored in future studies and its clinical application should consider short irrigation time as proposed for EDTA.

However, the irrigant solutions EDTA or GA did not negatively affected the dentin flexural strength, as reported by Bello *et al*.^14^. On the other hand, when comparing EDTA and GA with saline solution (control group), there were significant changes in the apatite/collagen ratio. These findings are agreement with others and support the hypothesis that the use of NaOCl prior EDTA and GA may promote collagen degradation and/or extraction and lower the apatite/collagen ratio^18^. Although is expected that this collagen degradation would also affect the mechanical property, the low concentration of NaOCl used in the present study (2.5%) might have resulted in less collagen breakdown, not enough to significantly reduce the flexural strength when compared to the control group, as previously suggested^19^. When comparing the FTIR spectra among the groups, the most significant changes are observed for the GA pH 5 (Fig. 1). Since aminomethyl propanol was used to obtain a GA solution with pH 5.0, this substance may have reacted with mineral and formed salts^20^, reflecting the significant changes in the apatite/collagen ratio found in this study. Similar spectra are noted for EDTA and GA pH 1.2, demonstrating a potential chelating binding of calcium mechanism for both solutions. However, it is important to mention that the IR spectrum is formed as a consequence of the absorption of electromagnetic radiation and conversion into molecular energy in the form of bands. Nevertheless, the depth of penetration of IR radiation in the ATR execution is in the order of a few microns^16^ and the FTIR results should be considered with caution.

Both EDTA and GA showed similar ability to remove smear layer. Recent studies showed that GA has the same ability to remove dentin smear layer than etching and irrigant solutions such as PA, EDTA and citric acid^11–13^. Citric acid has the ability to extract calcium ions from dentin, and is capable of removing the inorganic component of the smear layer and decalcify the dentin^13^. The acidic pH, low molecular weight, and organic nature of GA favor its use on mineral tissues. Given that citric acid is also an AHA, it can be suggested that GA and citric acid present similar mechanisms of action on cleaning the dentin surface. However, this hypothesis needs to be tested in further studies. All experimental solutions studied were less effective in removing the smear layer on the apical third of the root canal than on the coronal and middle thirds, corroborating with previous studies^13,21,22^. The broad diameter of the canal in the coronal and middle thirds exposes the dentin in these areas to a higher volume of the irrigant, thereby allowing a better flow and consequently making the region more approachable to cleaning procedures^21,22^. The ultrasonic agitation of EDTA increases its potential to remove the smear layer from the apical third^22^ and same results are expected for GA. Therefore, studies investigating agitation of GA inside of root canal should be carried out in the future.

Likewise, erosion scores were similar between EDTA and GA at both pH values in the coronal and middle thirds. The erosion caused on the wall of the root canal could modify some of the dentin properties^23,24^. The modification in the morphological components of dentin may be a contributing factor in the induction of dentin cracks^24^ and the alteration of the chemical constitution of underlying dentin might threaten the adhesion of root canal sealers^5^. The dentin erosion depends mainly on the pH and concentration of the solution, the time in contact with the dentin walls^18,24^, and the irrigation protocol used^24,25^. In the apical third, GA at higher pH had the same erosion scores as the control group. In previous studies, it was reported that dentin erosion was easily produced by irrigation with EDTA followed by NaOCl^24,26^. The main purpose of exposing the root canal dentin to EDTA is to remove smear layer and enable the direct contact of irrigating solutions with the dentinal walls. However, the effect of subsequent treatment with NaOCl on the chemical and ultramorphologic composition of root dentin results in the displacement of the collagen network and thinning of the fibrils, as well as extensive erosion of peritubular and intertubular dentin^24,25^. Therefore, the apparent aggressiveness of EDTA in causing root canal wall erosion is due to its use followed by NaOCl. Possibly, since NaOCl was used in all groups to simulate the clinical protocol of root canal therapy, it also promoted erosion when associated with GA.

In addition, no major differences were noted in the chemical composition of dentin when comparing the saline solution with the EDTA and GA groups, thus Ca and P contents remained stable. Na and Cl were detected in all groups, possibly associated with saline solution and NaOCl residue. A vestige of Mg was also found, which has been considered to influence the crystal growth in mineralization^21^. The root canal preparation was performed using the 2.5% NaOCl as irrigation solution and a sequence of three rotary instruments from the ProTaper Next system. This preparation protocol is fast and allows a shorter contact time between NaOCl and dentin. In addition, EDTA and GA were used for 1 minute inside the root canals. Thus, all the solutions used in this study remained for a short period of time in contact with dentin. This may justify the similar mineral content of dentin observed in all groups.

In conclusion, our results support the use of GA at neutral or acidic pH as an alternative irrigant in the root canal therapy. GA has the ability to remove the smear layer without promoting more erosion or negative effects on dentin mechanical properties. The use of GA at a lower pH seems to promote less change in collagen/apatite ratio, but future studies should clarify the effects of the pH on other dentin properties for the development of an ideal clinical protocol.

## Material and Methods

### Experimental design

The protocols and experiments proposed in this study were approved by the Ethics Research Committee of the University of Passo Fundo (No.#2.080.284) and teeth collection were performed in accordance with relevant guidelines and regulations as per the ethical and research board committee instructions. All teeth were obtained from the teeth biorepository of the School of Dentistry of the University of Passo Fundo under informed consent of the patients. After the extraction, the teeth were stored at 4 °C in 0.9% NaCl solution and were used within 1-month period.

For the treatment of dentin samples, the GA (Natupharma, Passo Fundo, Rio Grande do Sul, Brazil) was used at pH 1.2 and pH 5.0. To obtain GA solution at higher pH (5.0), a neutralizing agent, aminomethyl propanol, was used. As a positive control, 17% EDTA (Biodinamica, Ibipora, Parana, Brazil) was selected as the conventional final endodontic irrigant. Saline solution was used to treat samples in the negative control group.

The dentin surface demineralization was indirectly assessed by the microhardness test. The smear layer removal capacity and dentin erosion were evaluated by analyzing images obtained with Scanning Electron Microscopy (SEM). The analysis of mineral dentin distribution was assessed using Energy-Dispersive X-ray Spectroscopy (EDS). The apatite/collagen ratio and flexural strength of mineralized dentin were evaluated by Fourier-transform infrared spectroscopy (FTIR) and flexural strength test, respectively.

### Dentin microhardness

Twenty canine human teeth were sectioned transversely below the cementum-enamel junction producing 15-mm-long root segments. Thereafter, the roots were sectioned longitudinally in two, creating 40 specimens from the buccal and lingual segments. Each root specimen was embedded in self-curing acrylic resin, leaving the root canal dentin exposed on the surface. The dentin surfaces were then flattened using silicon carbide paper (500, 800, 1000, and 1200 grit) under constant water irrigation, and polished using a suspension of 0.1-mm alumina on a rotating felt disc.

In the negative control group (10 root segments), dentin surfaces were irrigated only with a saline solution. Dentin specimens in the other three groups (30 root segments) were irrigated with 2 ml of 2.5% sodium hypochlorite (NaOCl) for 1 min, followed by irrigation with 5 ml of saline solution and randomly divided according to the final irrigant (n = 10): 17% EDTA, 17% GA at pH 1.2, and 17% GA at pH 5. All final irrigations were performed with 2 ml of solution for 1 min. Finally, all samples were rinsed with 5 ml of saline solution.

Dentin microhardness was measured using a Knoop indenter with 40× magnification (SHIMADZU HMV-2000; Shimadzu Corporation, Kyoto, Japan) under a 25-g load and a dwell time of 15 s. Three indentations were performed on each specimen along lines parallel to the edge of the root canal in the apical direction. The first indentation was performed at 1.000 µm distance from the entrance of the root canal, and the other two indentations were performed at a distance of 200 µm from each other^15^. The values were averaged to generate one hardness value per specimen. Microhardness data showed normal distribution using the Shapiro-Wilk Normality and Equal Variance tests (p = 0.106 and p = 0.289, respectively). Data were statistically analyzed using one-way analysis of variance (ANOVA) and Tukey’s post-hoc test for multiple comparison (α = 0.05).

### Evaluation of smear-layer removal and dentin erosion

The chemical-mechanical preparation of 40 mandibular first premolar roots was performed using nickel-titanium rotatory instruments (ProTaper Next; Dentsply Maillefer, Ballaigues, Switzerland) following the manufacturer’s recommendations. The sequence of files used was X1, X2, and X3 at a speed of 300 rpm and torque of 2 N until the working length was achieved. At each change of instruments, the root canal was irrigated with 2 ml of 2.5% NaOCl. Thereafter, the 40 roots were randomly divided into four groups and the final irrigation was performed with 2 ml of the experimental solutions as previously mentioned. The solutions were applied inside the root canal as follows: 29-gauge needles (NaviTip Tips, Ultradent Products Incorporated, South Jordan, UT, USA) were inserted up to 3-mm short of the working length and the test solution was introduced until it fully filled the root canal. The test solution was inserted using up-down movement and remained within the root canal for one minute. Final irrigation was made with 5 ml of saline solution, and the canals were dried using absorbent paper cones^21^.

Thereafter, the 40 roots were divided into two halves resulting in 80 halves (20 in each group). The specimens were dehydrated using increasing concentrations of ethanol (25% for 20 min, 50% for 20 min, 75% for 20 min, 95% for 30 min, and 100% for 60 min). Each half root was mounted on metal stumps, covered with palladium gold (Quorum, Laughton, East Sussex, UK), and examined using SEM (VEGA LM 3; Tescan, Libušinatř. Kohoutovice, Czech Republic). Images were obtained to observe the morphology of the surface of the canal wall at 2000× magnification and 20 kV along the coronal (10–12 mm from the apex), middle (6–7 mm from the apex), and apical (1–2 mm apex) thirds of each specimen^21^. Two blinded independent investigators analyzed the presence or absence of the smear layer and verified root dentin erosion on the respective areas. The Kappa coefficient test showed a high agreement between the investigators regarding the interpretation of erosion scores (k = 0.876).

For the smear layer analysis the following criteria was used^27^: *Score 1:* no smear layer, dentinal tubules wide open; *Score 2*: small quantity of smear layer, some dentinal tubules open; *Score 3*: homogenous smear layer covering the root canal wall, only few dentinal tubules open; *Score 4*: entire root canal wall covered by a homogenous smear layer, no open dentinal tubules; and *Score 5*: heavy, non-homogenous smear layer covering completely the root canal wall. The images were classified according to the following specification for dentin erosion^23^: *Score 0*, smear layer covering almost all dentin surface, with few or no open tubules; *Score 1*, no erosion: all tubules without alteration in appearance and dimension; *Score 2*, moderate erosion: the peritubular dentin was eroded; and *Score 3*, strong erosion, the intertubular dentin was destroyed and the tubules were connected to each other.

Smear layer and dentin erosion data were determined to be non-parametric based on the Shapiro-Wilk Normality test (p<0.001). Therefore, data were analyzed with Kruskal-Wallis test and Dunn´s Method (α = 0.05), and results were presented using medians and quartiles (to represent data dispersion).

### Evaluation of mineral content by energy-dispersive X-ray spectroscopy (EDS)

The same specimens prepared in the previous test (smear layer removal and erosion) were used for the evaluation of surface mineral content. The entire area of the dentin matrix was visualized using SEM at a standard magnification of 200× (650×420 µm), and analyzed using EDS to determine the atomic ratio (at. %) of calcium (Ca), phosphorus (P), sodium (Na), chlorine (Cl), and magnesium (Mg). Changes in the mineral levels were recorded and differences among the groups were qualitatively analyzed.

### Apatite/collagen ratio using Fourier-transform infrared spectroscopy

Twenty non-carious extracted human mandibular third molar teeth were selected for this test. Enamel and cementum were removed from the teeth using a diamond bur #2215 in a high-speed handpiece under refrigeration. Dentin powder (90 µm) was obtained with a high-speed handpiece and diamond bur #3145 F without refrigeration. The powder went through a 90 µm sieve, so the dentin grains were equal to or smaller than this size. The dentin powder was divided in four groups of 9 mg each. One group remained untreated (NT), and the other three groups were rinsed with the GA and EDTA solutions. For irrigation, dentin powder was placed over a filter paper and fixed on a glass Becker. Irrigation was performed with 5 ml of the experimental solutions using a 25×4 mm needle for 1 min. After irrigation, the powder was washed three times with 5 ml of deionized water to remove residue of the experimental solutions and air-dried at 37 °C^13^.

FTIR spectra of the dentin powder were collected for each group (n = 3). Spectra were obtained between 650 and 4000 cm^−1^ resolution, using 48 scans (Agilent Cary; 630 FTIR spectrometer, Santa Clara, USA). The IR spectrum is produced as a result of the absorption of electromagnetic radiation at frequencies that correlate to the vibration of chemical bonds within a molecule. Thereby, when IR radiation is absorbed by an organic molecule, it is converted into molecular vibration energy, and the spectrum portray the vibrational motion and usually appears in the form of bands^28^. The range for the characterization of organic compounds is mid-IR (4000 to 400 cm^−1^)^29^.

Apatite/collagen ratios derived from FTIR spectroscopy showed normal distribution using the Shapiro-Wilk Normality test (p = 0.882) and Equal Variance test (p = 0.419). Data were statistically analyzed using one-way ANOVA and Tukey’s post-hoc test for multiple comparison (α = 0.05).

### Flexural strength test

Twenty human mandibular third molars were used for the flexural strength test. Mid-coronal dentin disks were cut perpendicular to the longitudinal axis of each tooth with a slow-speed diamond saw under constant water-cooling. The disks were trimmed to a final rectangular-shaped beam (5.0 mm length, 0.2 mm thick, 2.0 mm wide)^13^. Two beams were obtained from each tooth, totaling 40 beams, which were randomly divided into four groups (n = 10) following the same irrigation protocols previously mentioned.

Flexural tests were conducted using a three-point flexure device with a 3-mm support span. The specimens were tested at a crosshead speed of 0.5 mm/min using a universal testing machine (Instron, Canton, Norwood, USA). Flexural strength (in MPa) was calculated using the following equation: 3PL/2bd^2^, where P = load at fracture (N), L = length of support span (mm), b = beam width (mm)^13^.

Flexural strength data was normally distributed as observed in the Shapiro-Wilk Normality test (p = 0.05) and Equal Variance test (p = 0.405). Data were statistically analyzed using one-way ANOVA and Tukey’s post-hoc test for multiple comparison (α = 0.05).
